# Pembrolizumab-Responsive Cerebellar PML in Untreated Sarcoidosis: A Case Report

**DOI:** 10.1007/s12311-026-01973-9

**Published:** 2026-02-28

**Authors:** Ella Jack-kee, Natasha Hoyle, Marios Hadjivassiliou

**Affiliations:** 1 Medical School, The University of Sheffield, Sheffield, UK; 2 Sheffield Teaching Hospitals NHS Foundation Trust, Royal Hallamshire Hospital, Glossop Road, Sheffield, S10 2JF UK

**Keywords:** Progressive Multifocal Leukoencephalopathy, Sarcoidosis, Cerebellar Peduncles, Ataxia, Pembrolizumab, Case Report

## Abstract

Progressive multifocal leukoencephalopathy (PML) is a rare but devastating demyelinating disease usually seen in immunocompromised patients. We describe a 52-year-old man with pulmonary sarcoidosis, who presented with progressive ataxia, dysarthria, and unsteadiness of gait. He was not on immunosuppressive therapy at the onset of balance problems. Initial imaging showed increased signal in the cerebellar peduncles suggestive of cerebellar variant of multiple system atrophy (MSA-C). Cerebellar biopsy revealed PML. The patient’s condition deteriorated until pembrolizumab, a PD-1 checkpoint inhibitor, was commenced. He subsequently showed slow but sustained clinical improvement, supported by radiological (reduced enhancement) and spectroscopic evidence (increased N-Acetyl Aspartate to Creatine ratio), and he remains stable four years later. This case highlights the diagnostic challenge of PML in the absence of ongoing immunosuppression, and illustrates the potential role of pembrolizumab as a disease-modifying therapy. Early recognition and consideration of immunotherapy may be critical to improving outcomes in this otherwise often fatal condition.

## Introduction

Progressive multifocal leukoencephalopathy (PML) is a demyelinating condition of the central nervous system caused by the reactivation of the JC virus (JCV), resulting in high mortality rates among those infected [1]. PML typically presents in immunocompromised patients, most commonly with acquired immune deficiency syndrome (AIDS). However, more cases are being seen with lympho/myeloproliferative disorders, autoimmune diseases on treatment with immunosuppression (e.g., multiple sclerosis), and iatrogenic immunosuppression following a transplant [2]. The mechanism behind the reactivation of the JCV remains largely unclear, but the host’s immune response is thought to contribute to the development of PML, as this viral reactivation tends to be more common in patients with CD4 + T cell depletion.

Sarcoidosis is an autoimmune, systemic, granulomatous disorder characterized by the presence of non-caseating epithelioid granulomas, primarily affecting the lungs and lymph nodes [1]. Patients presenting with both sarcoidosis and PML typically are undergoing immunosuppressive treatment, such as with corticosteroids [3].

More rarely, cases have been reported of patients developing PML following untreated sarcoidosis [4], possibly making sarcoidosis a predisposition to PML, and the association between the two may not be due to the treatment alone. The evidence supporting this remains limited; however, it is hypothesised that active systemic sarcoidosis may lead to an elevated frequency of regulatory T-cells, alongside a concomitant reduction in CD4 + T-cell counts [1]. These immunological alterations could potentially predispose sarcoidosis patients to the development of PML.

Whilst there is no standardised treatment for PML in most cases, recent advances have introduced some promising disease-modifying interventions. One treatment currently under review for PML is pembrolizumab, a programmed cell-death-1 (PD-1) immune checkpoint inhibitor [2]. PD-1 expression has been observed to be elevated on CD4 + and CD8 + cells in patients with PML, with a notable increase on JC virus-specific CD8 + cells. When the PD-1 receptor binds to its ligands, the cytotoxic T cell response is inhibited. Pembrolizumab enhances T cell activation by inhibiting this interaction, potentially posing therapeutic benefits for PML.

We present a case of a patient with sarcoidosis-associated PML, who showed clinical improvement following pembrolizumab treatment.

## Case Description

A 52-year-old male presented in September 2020 with unsteady walking and slurred speech. He had noticed some slurring of speech since early 2019, but it was the unsteadiness of gait that prompted the referral to the local hospital. His medical history included pulmonary sarcoidosis, diagnosed in 2015 and previously managed with prednisolone 10/5 mg on alternate days; however, he was not on any steroids or other treatment for a whole year before the onset of his balance problems.

**Fig. 1:**
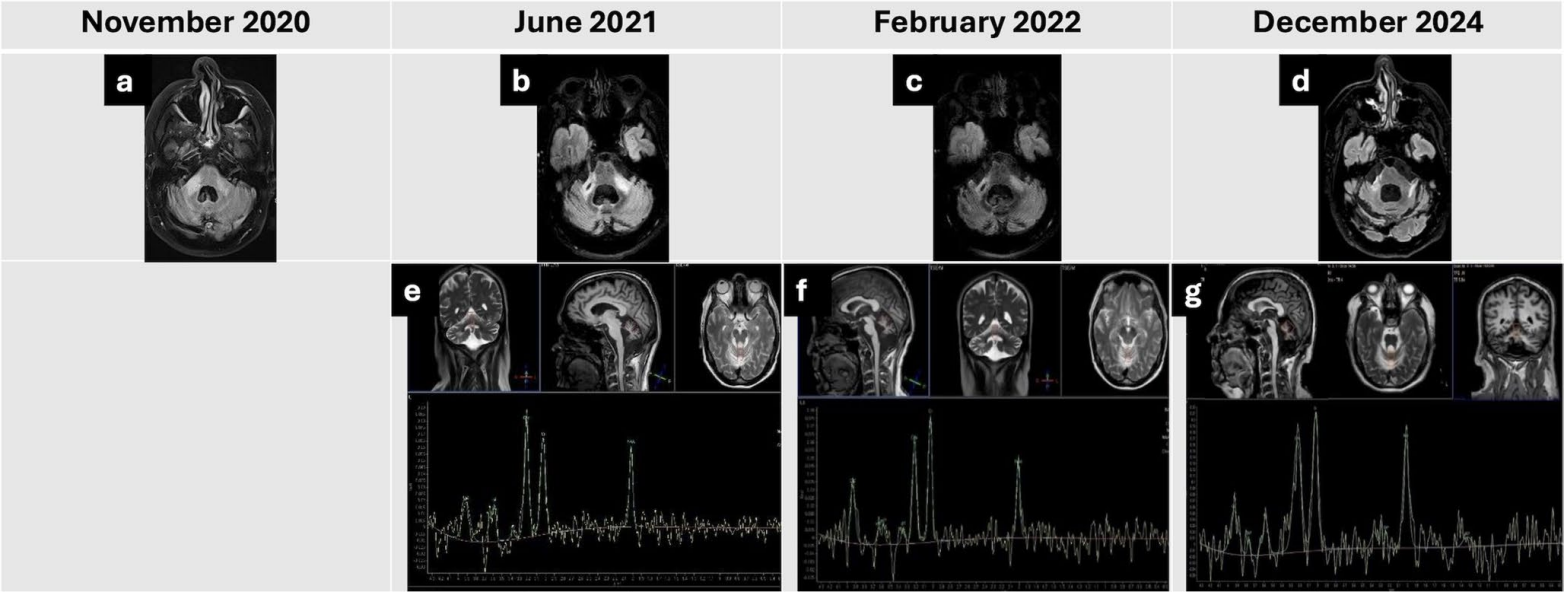
Serial MRI and MR spectroscopy findings

The patient’s brain MRI in November 2020 was reported to show increased signal in the cerebellar peduncles (Fig. 1a). Repeat MRI with contrast showed no enhancement. Cerebrospinal fluid (CSF) analysis at this time revealed slightly elevated protein levels (0.9 g/L) and positive oligoclonal bands. These MRI findings, alongside the relatively rapidly progressive ataxia and early speech involvement, led to the possible diagnosis of the cerebellar variant of multiple system atrophy (MSA-C) by the local medical team that saw him initially, although the positive oligoclonal bands were not compatible with such a diagnosis. In addition, the patient had no autonomic features. A whole-body PET scan was normal.

The MRI images were sent for review at the Sheffield Ataxia Centre by another neurologist who had concerns about the validity of the diagnosis of MSA-C. On review of the imaging, it was felt that there was striking asymmetry and somewhat nodular appearances of the high signal within the cerebellar peduncles, which are not typical of what is seen in MSA-C. After discussing this with the local neurologist, a decision was made to proceed with a biopsy of the affected area. A biopsy of the right cerebellar peduncle confirmed the diagnosis of PML in December 2020. Laboratory investigations at this time revealed persistent CD4 lymphopenia, with an absolute CD4 + T-cell count of 0.18 × 10⁹/L (180 cells/µL). Investigations looking at the causes of an immunocompromised state were normal.

The patient lived alone, with carers assisting twice daily. He was a non-smoker, did not consume alcohol, and, due to worsening dysarthria, communication was done through an iPad or email.

Over the following six months, his condition deteriorated, the dysarthria worsened, exercise tolerance diminished, and he experienced significant weight loss.

The patient was reviewed at the Sheffield Ataxia Centre in June 2021. Neurological examination showed severe dysarthria but no nystagmus. He had finger-nose ataxia and more prominent heel-to-shin ataxia. He had severe gait ataxia and was unable to walk without the help of at least one person. His Scale for the Assessment and Rating of Ataxia (SARA) was 27.

MRI in June 2021 showed increased bilateral signal intensity in the cerebellar peduncles with associated volume loss (Fig. 1b) and markedly abnormal MR spectroscopy (Fig. 1e). Blood tests revealed elevated urea (15.4 mmol/L), elevated creatinine (189 µmol/L), and reduced lymphocytes (0.44 × 10⁹/L). Hepatitis B, C, and HIV serologies were negative. Lumbar puncture for JCV was negative. Serum immunoglobulins were normal.

As pembrolizumab in the treatment of PML had been reported in the literature, following further discussions with the patient and local approval, the patient was admitted to the Sheffield Ataxia Centre for pembrolizumab treatment. This was started in August 2021 (150 mg, two doses one month apart). The patient demonstrated slow but sustained clinical improvement (SARA score after the first infusion was 25), with no new symptoms; the only reported side effect was fatigue. Speech, however, remained markedly hypophonic, and communication continued via iPad.

At six months, MRI showed reduced signal intensity in the cerebellar peduncles (Fig. 1c), with corresponding improvement in cerebellar spectroscopy (Fig. 1f). After three years, spectroscopy demonstrated marginal but sustained further improvement (Fig. 1g), accompanied by near-complete resolution of hyperintense lesions in the left cerebellar peduncle on MRI (Fig. 1d).

The patient remains stable, with no suggestion of any progression four years after the one-off treatment with pembrolizumab. He has, however, been left with significant disability as a result of the cerebellar involvement. He still has a slight lymphopenia (0.49, range 1-3 × 10^9^/L) but normal immunoglobulins.

## Discussion

This case report demonstrates a patient with PML in association with sarcoidosis, who showed clinical improvement following pembrolizumab treatment. Only one other similar case has been reported [5]. In that case, the patient’s condition worsened despite steroid treatment, intravenous immunoglobulin (IVIG), and mirtazapine. Pembrolizumab treatment was commenced, and clinical findings improved after the fourth dosage. Negative JCV polymerase chain reaction (PCR) was found in the CSF 7 months after starting treatment, and the patient completed 8 doses of pembrolizumab with no adverse side effects. Later MRI neurological assessment indicated substantial resolution of the lesion, although atrophy of the cerebellum and cerebellar peduncle, predominantly on the right side, was observed. Whilst both this case and our case show clinical improvement following pembrolizumab treatment, more research is required to consolidate pembrolizumab as a treatment option for sarcoidosis-associated PML. The timeliness of pembrolizumab treatment may also affect clinical outcomes. Having a sufficient number of functional lymphocytes prior to commencing treatment has been positively linked to pembrolizumab’s success [6]. This may indicate the need for early diagnosis and intervention to improve outcomes for pembrolizumab treatment in PML.

Furthermore, this case highlights the vulnerability of the cerebellar peduncles to JC-virus–mediated demyelination. JC virus exhibits marked tropism for oligodendrocytes, leading to patchy but destructive demyelination within heavily myelinated white-matter tracts, such as the cerebellar peduncles [7]. Disruption of these major afferent and efferent pathways can generate a clinical picture closely resembling MSA-C when lesions are bilateral and progressive. However, the asymmetry and nodularity seen in our patient diverge from the imaging patterns typically associated with cerebellar neurodegeneration and instead reflect the multifocal, irregular pattern of JC-virus-driven oligodendroglia injury [1, 4]. The subsequent radiological improvement following pembrolizumab further supports an immune-mediated, rather than degenerative, cerebellar process. This case therefore reinforces the importance of considering inflammatory, demyelinating, or infectious pathology when cerebellar peduncle abnormalities are not associated with a clinical picture supportive of MSA-C.

Other therapies have been used to treat PML in sarcoidosis patients. Despite its immunosuppressive properties, infliximab, a tumor necrosis factor-alpha inhibitor, has shown potential therapeutic benefit in improving PML prognosis [1]. In a cohort study, three patients with PML and sarcoidosis showed continuous clinical improvement of symptoms under infliximab treatment, with undetectable levels of JC virus in the CSF and decreased regulatory T-cell frequencies. Cidofovir has also demonstrated potential efficacy as a treatment. In a reported case of sarcoidosis-associated PML, treatment with cidofovir alone resulted in neurological and radiological stabilisation, without PML relapse after more than two years off-treatment [8]. Another case report described a near-complete recovery and significant symptomatic improvement following combination therapy with cidofovir and mirtazapine, highlighting the potential benefit of dual treatment [9].

Pembrolizumab has also been used to treat PML in HIV patients. In a study of five HIV-positive patients with PML, a JCV DNA decline was observed in all patients following pembrolizumab treatment, with three out of five patients displaying progressive reduction in the size of the cerebral lesions on MRI [10]. A separate study reported successful pembrolizumab treatment in two HIV-associated PML patients. In one patient, motor aphasia and left arm weakness improved with 2 doses of pembrolizumab, with clearance of JCV from CSF; the second HIV patient experienced symptom and JCV load fluctuation, but after 3 doses of pembrolizumab, showed clinical and radiological stabilisation and a decline in JCV load - yet, the association between degrees of symptoms and treatment remained unclear [7]. A case report of a 64-year-old male with HIV and PML showed no response to pembrolizumab, showing no improvement in left hemiparesis and developing left hemineglect, before eventually dying - although the patient was reported to have an advanced stage of HIV/AIDS at presentation [2].

## Conclusions

This case report highlights the rare but clinically significant association between sarcoidosis and PML, even in the absence of ongoing immunosuppressive therapy. The patient demonstrated sustained clinical and radiological improvement following treatment with pembrolizumab. Whilst the use of pembrolizumab in PML remains investigational, this case adds to the growing body of evidence supporting its potential therapeutic role. Given the high mortality and limited treatment options for PML, further research into immunotherapeutic options and treatment timeliness is needed to establish optimal management protocols for PML, particularly those sarcoidosis-associated.

**Axial T2-Fluid-attenuated inversion recovery (FLAIR) image of the brain of the patient**:

- Two months after presentation with ataxia, showing increased signal in the cerebellar peduncles, more prominent on the right.
- Nine months after presentation (untreated, post-biopsy), showing increased signal in both cerebellar peduncles with bilateral volume loss, and additional atrophy of the fourth ventricle and brainstem.
- Six months after pembrolizumab treatment, showing reduced signal intensity in the cerebellar peduncles.
- Three years after pembrolizumab, showing marked improvement with further reduction of signal intensity bilaterally.

**MR spectroscopy of the superior cerebellar vermis**:

e. Nine months after presentation (untreated), showing a markedly reduced N-acetyl aspartate to creatine (NAA/Cr) ratio of 0.58 (normal > 1).
f. Six months after pembrolizumab, showing NAA/Cr ratio of 0.62.
g. Three years after pembrolizumab, showing significant improvement with an NAA/Cr ratio of 0.82.

## Data Availability

All data relevant to the case are included within the article.
